# Preservation of orbit in tumor invasion through the periorbita in sinonasal malignancy

**DOI:** 10.1007/s00405-024-08757-z

**Published:** 2024-06-24

**Authors:** Stepan Novak, Zuzana Balatkova, Alzbeta Fikova, Marek Grega, David Kalfert, Jan Plzak

**Affiliations:** 1 Department of Otorhinolaryngology and Head and Neck Surgery, First Faculty of Medicine, University Hospital Motol, Charles University, V Uvalu 84, Prague, 150 06 Czech Republic; 2 Department of Pathology and Molecular Medicine, Second Faculty of Medicine, Charles University, University Hospital Motol, Prague, Czech Republic

**Keywords:** Sinonasal malignancy, Periorbital infiltration, Orbital exenteration, Orbital preservation, Survival, Vision

## Abstract

**Objective:**

One of the possible risks of sinonasal malignancy is its possible spread in the orbit. However, there is no clear consensus among the different departments as to whether it is necessary to exenterate the orbit in limited tumorous infiltration of periorbital fat. The purpose of the study was to demonstrate that periorbital infiltration and periorbital fat invasion without involvement of deeper orbital tissues are not the indication of orbital exenteration.

**Materials and methods:**

Retrospective analysis was performed over a 17-year period of patients undergoing surgical treatment for sinonasal malignancy with histologically verified periorbital infiltration or deeper invasion into the orbit. A total of 32 patients were included in the study. For each group, the following data were analysed: sex, age, preoperative imaging studies, histological findings, site of origin, stage, surgical reconstruction, oncological treatment, survival, cause of death, number of recurrences in the orbit and functional status of preserved eyes.

**Results:**

Based on our criteria for orbital exenteration, orbital preservation was feasible in 18 patients. Orbital exenteration was performed in 14 patients with deeper tumor infiltration. There was a statistically insignificant difference in survival between the two groups. The 5-year overall survival (OS) was 44% for the orbital preservation group (only 2 patients died from local tumor recurrence) and 34% for the orbital exenteration group. The groups did not differ in other observed factors other than the extent of orbital infiltration. In 11 (61.1%) patients, vision was without significant change after radiation therapy. In 2 (11.1%) patients, visual function was impaired due to diplopia. 5 (27.8%) patients had severely impaired vision due to optic nerve atrophy after radiation therapy.

**Conclusions:**

Our results show a relatively high survival rate in the group of patients with orbital preservation with a high chance of vision preservation, which justifies our approach to orbital preservation even in some tumors with periorbital infiltration.

## Introduction

It is well known that orbital invasion in sinonasal cancers is associated with poor prognoses for overall survival [1, 2] and the involvement in orbital soft tissue is an independent factor that significantly influences the survival [3]. Several case series worldwide have supported evidence that the progressive involvement of orbital structures decreases 5-year OS from 65%-55–20-30% [4, 5]. In the past, tumors were generally treated with higher radicality regardless of functional results. This condition was gradually modified due to a better understanding of tumor biology and due to more sensitive imaging methods, more perfect surgical tools, and finally, thanks to more perfectly targeted RT. In the 1970s, orbital exenteration was recommended in all cases with bony orbital invasion [6, 7]. However, even such an aggressive and mutilating surgery had a limited impact on the prognosis of the disease [8]. Gradually, the indications for orbital exenteration have evolved. However, the impact of orbital exenteration or preservation on survival is still unclear, and indications for orbital exenteration have always been controversial [4]. Various indications for orbital exenteration have been proposed, based on invasion of the orbital wall [9], transgression of the periosteum [10], orbital fat involvement, extraocular muscle, ocular globe, orbital apex, or eyelid invasion [11–13]. In the work of Iannetti et al. [12] three stages of orbital invasion are defined: grade I, erosion or destruction of the bony orbital wall; grade II, extraconal invasion of the periorbital fat; and grade III, invasion of the medial rectus muscle, optic nerve, ocular bulb, or skin overlying the eyelid. This rating scale can be used for comparison between individual studies. In 2015, the meta-analysis compared 4 large retrospective studies of SNC infiltrating the orbit [4]. Wu et al. [9] used grade I, Lund et al. [10] used grade II, Imola et al. [11] and Ianneti et al. [12] used grade III as an indication of exenteration. In the work of Lisan et al. [13], the indication for orbital exenteration was the invasion of one or more of the following: extraocular muscles, ocular globe, or orbital apex. Shin et al. [14] recommend exenteration as early as orbital fat invasion. In the recent work of Turri Zanoni et al. survival in the case of orbital apex infiltration was extremely poor, regardless of the extent of surgery [8]. Based on these data, exenteration of the orbit is not indicated in that case [8].

The concept of selective orbital preservation surgery with adjunctive preoperative radiation for paranasal sinus malignancy was introduced. This method allowed preservation of the orbit with satisfactory survival [15, 16]. The modern trend in poorly differentiated tumours is neoadjuvant chemotherapy which, depending on their response, allows orbital preservation in a large group of patients [8].

If the orbit is preserved, the functionality of the eye is also questionable. In such advanced tumors, adjuvant radiotherapy is almost always indicated, with an additional risk of vision deterioration [12].

The aim of the study was to investigate that periorbital infiltration with periorbital fat invasion of the tumor without involvement of deeper orbital tissues is not an indication criterion for orbital exenteration.

## Materials and methods

The retrospective study was conducted over a 17-year period (2005–2022) in patients undergoing surgical treatment for SNC with histologically verified periorbital infiltration, orbital fat involvement, extraocular muscle invasion or eyelid invasion. All patients underwent surgery in the Department of Otorhinolaryngology and Head and Neck Surgery, 1st Faculty of Medicine, Charles University, and University Hospital in Motol in Prague. Patients with orbital bone invasion without periorbital infiltration were not included in the study. Our indications for orbital exenteration included: (1) involvement of the orbital apex or retrobulbar fat, (2) extension into the extraocular eye muscles, (3) extension into the intraconal space of the orbit, (4) invasion of the eye bulb or optic nerve, and (5) lid involvement. The orbit was preserved in all tumors infiltrating through the periorbita, but at the same time did not meet the indication criteria for orbital exenteration. All patients underwent a preoperative computer tomography (CT) or magnetic resonance imaging (MRI) examination (or both), which offers valuable preoperative information. The final decision on the orbital management was made intraoperatively (on the basis of macroscopically findings during surgery and intraoperative frozen sections) and only patients with histologically verified periorbital infiltration were included in the study. A total of 32 patients were included in the study. The orbit was preserved in 18 patients and orbital exenteration was performed in 14 patients.

For the histological classification was used WHO Classification of Tumours edition according to the time of diagnosis. Patients in our study were chosen from a long-time period, so the applied classification includes several editions (3rd ed., 4th ed., 5th ed.).

Tumors were completely removed macroscopically in all cases. Intraoperative frozen section was performed in all patients to achieve adequate margins. Final histopathology was assessed as R0 in all patients. The stratification into the groups was based on orbital exenteration or preservation. For patients in each group, the following data were searched: sex, age, preoperative imaging studies, histological findings, site of origin, stage, prior treatment, surgical reconstruction, adjuvant oncological treatment, survival, cause of death, the number of recurrences in the orbit and functional status of the preserved eye (in case of orbital preservation). Eye function was assessed based on daily use of the eye, changes in quality of vision, and diplopia. Due to the negative long-term effect of adjuvant radiation therapy, ocular function was evaluated based on the patient’s last documented outpatient visit. Patients with severe subjective or objective findings were further evaluated and treated by an ophthalmologist.

### Statistical analysis

The IBM SSPS version 22.0 statistical program was used for the statistical processing of the results. Descriptive statistics methods, Fisher’s exact test (two-sided) and Pearson’s chi-square test were chosen. The Kaplan-Meier method was used to assess overall survival and survival based on various variables. A value of *p* < 0.05 was chosen to assess statistical significance.

## Results

### The orbital preservation group

It consisted of 6 women and 12 men. The age ranges from 32 to 78 years with a mean age of 56.9 and a median age of 56.5 for all patients. For men the range is 32 to 78 years with a mean age of 57.6 years and a median age of 58.5. The range for women is 44 to 75 years with a mean age of 55.7 and a median age of 50.

The tumor histology and the site of origin are shown in Table 1. The most common histological type was sinonasal undifferentiated carcinoma (SNUC) in 9 patients. Other histological types were squamous cell carcinoma (SCC), adenoid cystic carcinoma (ACC), and sarcoma.

**Table 1:** The tumor histology and the site of the origin for the orbital preservation and the orbital exenteration group

	Site of Origin	SNUC	SCC	ACC	Sarcoma	MEC
*orbital preservation*	ethmoid sinus	6	0	0	0	0
	maxillary sinus	0	3	2	1	0
	nasal cavity	2	1	0	0	0
	lacrimal apparatus	0	1	1	0	0
	frontal sinus	1	0	0	0	0
*orbital exenteration*	ethmoid sinus	0	1	1	1	2
	maxillary sinus	1	4	0	0	0
	nasal cavity	0	2	0	0	0
	lacrimal apparatus	0	2	0	0	0
	frontal sinus	0	0	0	0	0

SCC = squamous cell carcinoma; SNUC = sinonasal undifferentiated carcinoma; ACC = Adenoid cystic carcinoma; MEC = Mucoepidermoid carcinoma

The follow-up of the group ranged from 5 to 10 years. Preoperative CT of paranasal sinuses was performed in all patients and MRI was performed at the same time in 8 patients. PET/CT was performed in 4 patients. Whole-body CT was performed in 1 patient.

The TNM classification is summarized in Table 2. The most common tumor extent in 66.7% was T4aN0. Nodal metastases were present in 5.6%.

**Table 2:** TNM classification for the orbital preservation and the orbital exenteration group

TNM	orbital exenteration	orbital preservation
T4aN0	9	12
T4aN2b	1	1
T4bN0	4	5

2 patients underwent reoperation for recurrence of an initially smaller tumour. The surgical approach was through a lateral rhinotomy or Weber-Fergusson incisions in 12 patients. In 2 patients, the tumor could be removed endoscopically. In 4 patients, the nasal portion of the tumor was removed endoscopically, and the intracranial portion of the tumor was removed by a neurosurgeon from a bifrontal craniotomy.

In 8 cases, the orbit was not reconstructed. The postoperative defect was reconstructed by a fascia in 8 cases. The tensor fascia lata flap (TFLF) was used in 2 cases. The temporoparietal fascia flap (TPFF) was used in 6 cases. In 1 case, the fasciocutaneous free flaps of the radial forearm were used for reconstruction. LactoSorb material was used in 1 case.

The type of adjuvant oncological treatment in relation to the histological type of tumor is summarized in Table 3. Radiation therapy (RT) was performed in 12 patients. Chemoradiotherapy (CHRT) was performed in 4 patients. 2 patients did not undergo any further oncological treatment. In 1 patient, adjuvant oncological treatment was not indicated due to previous RT and in 1 patient adjuvant RT was indicated, but the patient did not undergo treatment due to a postoperative complication and rapid recurrence with subsequent death.

**Table 3:** The type of the adjuvant oncological treatment, divided on the basis of histological type of the tumors

		SNUC	SCC	ACC	Sarcoma	MEC
*orbital preservation*	RT	3	4	3	1	0
	CHRT	4	1	0	0	0
	No	2	0	0	0	0
*orbital exenteration*	RT	0	4	1	1	2
	CHRT	1	2	0	0	0
	No	0	3	0	0	0

SCC = squamous cell carcinoma; SNUC = sinonasal undifferentiated carcinoma; ACC = Adenoid cystic carcinoma; MEC = Mucoepidermoid carcinoma

RT = radiotherapy; CHRT = chemotherapy; No = without oncological treatment

### The orbital exenteration group

It consisted of 3 women and 11 men. The age ranges from 32 to 67 years with a mean age of 54.4 and a median age of 54.5 for all patients. The range for men is 32 to 67 years with a mean age of 55.5 and a median age of 55. For women, ranges from 44 to 55 years with a mean age of 50.3 and a median age of 52.

The follow-up of the group, in general, ranged from 5 to 10 years. Preoperative CT of paranasal sinuses was performed in all patients and MRI was performed at the same time in 8 patients. PET/CT was performed in 3 patients.

The TNM classification is summarized in Table 2. The most common extent of the tumor in 64.3% was T4aN0. Nodal metastases were present in 7.1%.

The tumor histology and the site of origin are shown in Table 1. The most common histological type in the number of 9 patients was SCC. Other histological types were SNUC, ACC, mucoepidermoid carcinoma, and sarcoma.

Primary oncological treatment preceded surgery in 2 patients. Neoadjuvant chemotherapy was indicated in 2 patients due to the extent of the tumor. In 3 patients, it was the reoperation for the recurrence of the initially smaller tumor. The surgical approach was through a lateral rhinotomy or Weber-Fergusson incisions in 13 patients. In 1 patient, the approach was made by facial translocation.

The resulting defect was not reconstructed in 3 cases, reconstructed by fascia in 5 cases (TFLF in 1 case, TPFF in 5 cases). For a more extensive reconstruction, the free flap was used in 4 cases (anterolateral thigh flap in all cases) and the pedicle flap (temporal muscle flap) in 1 case. In 2 patients, the intracranial portion of the tumor was removed by facial translocation or bifrontal craniotomy.

The type of adjuvant oncological treatment in relation to the histological type of tumor is summarized in Table 3. Adjuvant RT was performed in 8 patients. Adjuvant CHRT was performed in 3 patients. 3 patients did not undergo any further oncological treatment. In 2 patients, no oncological treatment was indicated due to previous CHRT for SNC. In 1 patient, adjuvant oncological treatment was not indicated due to previous CHRT for (duplicity of) an oropharyngeal cancer.

### Survival

**Fig. 1:**
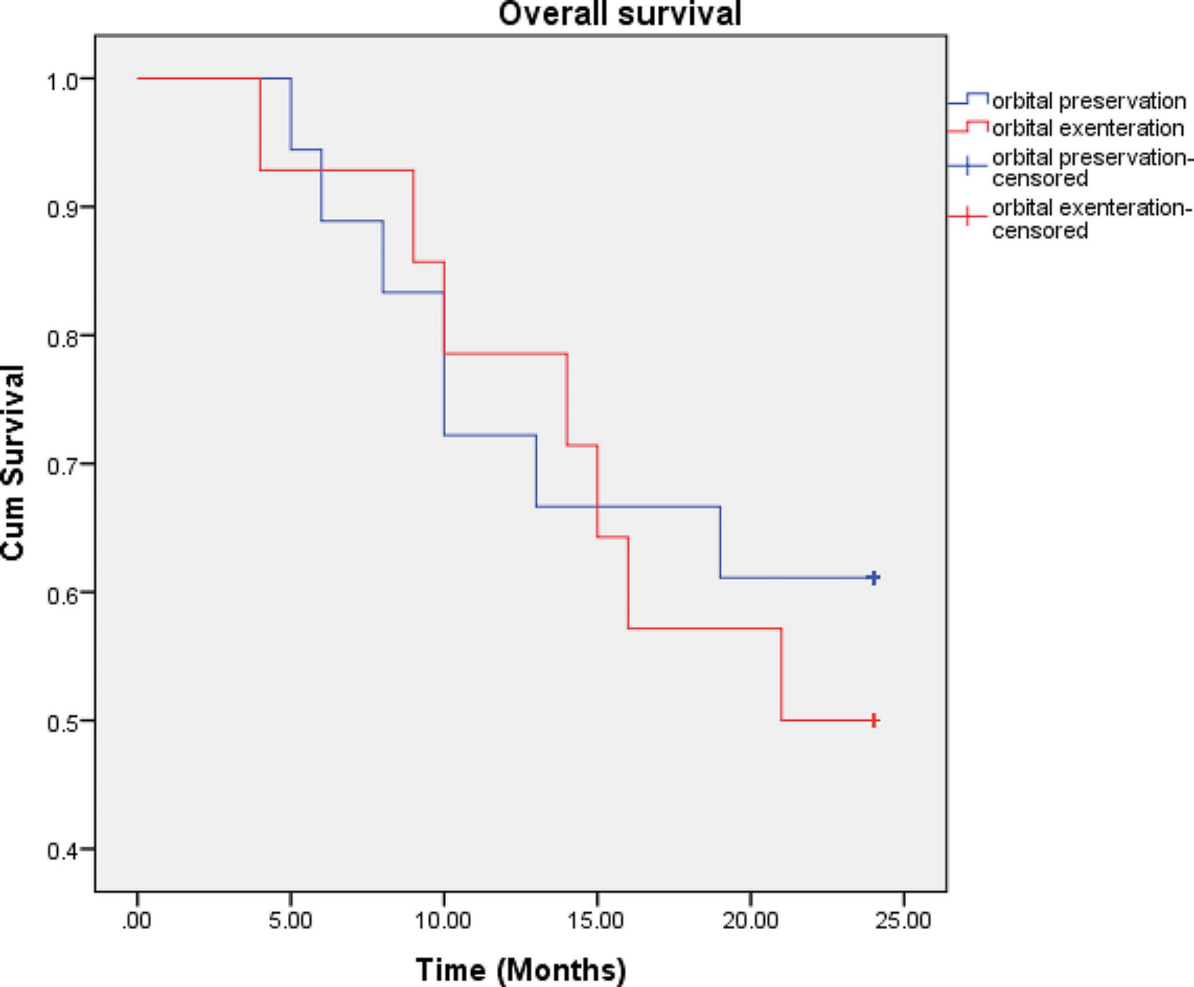
The overall survival curves. The 2-year survival in the orbital preservation group (18 patients) was 61% as compared with 50% in the group of patients who underwent orbital exenteration (14 patients). No statistical difference was seen between the two groups

**Fig. 2:**
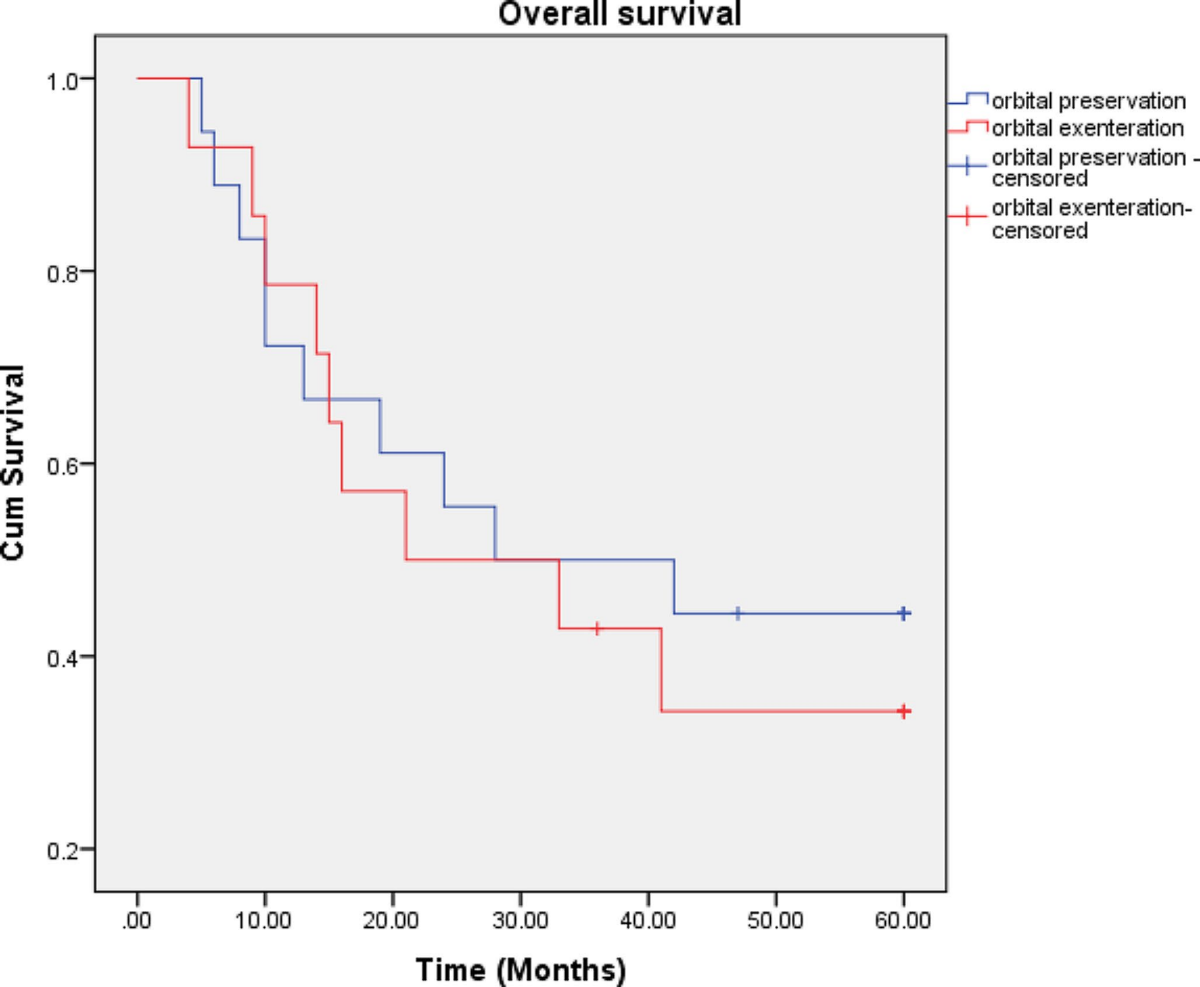
The overall survival curves. The 5-year survival in the orbital preservation group (18 patients) was 44% as compared with 34% in the group of patients who underwent orbital exenteration (14 patients). No statistical difference was seen between the two groups

The difference in 2- and 5-year OS between the two surgical procedures was not statistically significant. The 2-year OS was 61% for orbital preservation and 50% for orbital exenteration. The 5-year OS was 44% for orbital preservation and 34% for orbital exenteration. The 2-year and 5-year OS curves for orbital preservation and orbital exenteration are shown in Figs. 1 and 2.

There were no statistically significant differences in OS based on different histological types.

Tumor recurrence or type of adjuvant oncological treatment were not associated with statistically significant worse OS.

In the group with orbital preservation, only 2 patients (11%) were diagnosed with local recurrence with involvement of the original orbital site. The incidence of local recurrences is detailed in Table 4. Local recurrence occurred with similar frequency in both patients undergoing orbital exenteration and those with preserved orbit.

**Table 4:** The cause of the death during 5-year follow-up for the orbital preservation and the orbital exenteration group

5-year follow-up	orbital preservation	orbital exenteration
Survived	8	5
Local sinonasal recurrence +Orbital Disease	2	0
Local sinonasal recurrence -Orbital disease	5	3
Distant metastases	3	4
Unknown cause of death	0	2

- Orbital disease: local recurrence in the sinonasal cavity remote from the original site of orbital involvement

+ Orbital disease: local recurrence in the sinonasal cavity with involvement of the original orbital site

### Vision

In 11 (61.1%) patients, vision did not change significantly after radiation therapy. In this group of patients, epiphora or keratitis with early correction occurred and 4 patients underwent successful cataract surgery without subsequent visual impairment.

In 2 (11.1%) patients, visual function was impaired due to diplopia. In 1 patient, when the eye was moved laterally and in 1 patient, when the eye was moved in all directions.

5 (27.8%) patients had severely impaired vision due to optic nerve atrophy, 3 of them also had postoperative diplopia.

According to the classification described by Imola and Schramm [11] eye-related functional outcomes were the following: 61.1% grade I (functional without impairment); 11.1% grade II (functional with impairment); and 27.8% grade III (non-functional).

The histological type of the tumor or the type of oncological treatment had no effect on vision.

## Discussion

Our philosophy is that we try to preserve the orbit even in the case of invading the SNC to the orbital soft tissues with penetration through the periorbita. When using the scale according to Iannetti et al., the indication for orbital exenteration in our hands is grade III [12]. Previously, we considered infiltration of the orbital apex as the indication for the orbital exenteration. Based on the extremely poor prognosis of OS and the mutilation in patients undergoing extensive surgery, we would prefer the oncologic treatment [8].

The combination of CT and MRI is a relatively sensitive method that enables the evaluation of the extent of tumor invasion into the orbit, facilitating preoperative consultation with the patient about the realistic possibility of preserving the eye. Currently, we require preoperatively MRI in every patient with sinonasal malignancy. Due to the long-term retrospective study and the historically poorer availability of MRI in the Czech Republic, some patients did not have preoperative MRI.

However, the definitive decision about the extent of resection is made intraoperatively on the basis of macroscopically findings during surgery and intraoperative frozen sections. In some patients with larger tumors, PET/CT was chosen based on discussion at the multidisciplinary oncology team, although we do not have uniform criteria for its indication. Occult neck node metastasis was not found in any patient using this method.

In the orbital preservation group, SNUC occurred more often, although the difference did not reach statistical significance. Compared to other retrospective studies, there was more SNUC in our group of patients; on the contrary, no adenocarcinomas were found [4].

Currently, neoadjuvant chemotherapy (NAChT) is considered for advanced tumors of the following histologies: poorly differentiated SCC, Hyams grade III/IV olfactory neuroblastoma (ONB), SNUC, and neuroendocrine carcinomas (NECs) [8, 17]. These recommendations have been followed in our department only in recent years, therefore, these patients are not included in the study.

Despite the very advanced extent of the tumor, only 6% of the patients had nodal metastases. In both cases, multiple and one-sided. There are studies showing better regional control when elective neck dissection (END) is performed [18], but END is not routinely performed in most centres worldwide [17]. Similarly, END is not recommended in the latest NCCN [19]. In our department, END is not indicated even for advanced tumors. END is replaced by the inclusion of regional lymph nodes in the clinical target volume (CTV) of RT. This strategy allows for the treatment of even retropharyngeal or intraparotic lymph nodes that are not accessible to END. No isolated recurrence in regional lymph nodes was found in our study.

Our results did not show statistically significant differences in 2-year or 5-year overall survival rates between the two groups. Overall survival in the orbital preservation group is even better than in the orbital exenteration group, although it did not reach statistical significance. The 5-year survival rate of 44% for orbital preservation and 34% for orbital exenteration is comparable to other retrospective studies [4, 13, 14]. It should be emphasized here that only patients with tumor invasion through the periorbita were included in our cohort of patients. In the other studies, patients with tumor infiltration of the bony walls of the orbit and thus with less advanced tumors were also included [4, 9–13].

We consider the relatively low local recurrence (10.5%) with involvement of the original orbital site to be very important.

Sometimes, the limited value of such a comparison is stated due to the inherent substantial selection bias inherent [11]. Patients subjected to orbital exenteration fall into a more advanced disease group with a worse prognosis compared to patients in whom orbital preservation is an option. On the other hand, it is necessary to state that the extent of infiltration of the orbit is not the only criterion determining the extent of the tumor and, for example, in 53% of patients in the orbital preservation group, the skull base was infiltrated, compared to 50% in the orbital exenteration group. This makes the conclusions regarding the comparison of tumor extent more complicated.

The most aggressive histological type of the tumors in our study was the SCC. However, the difference in the survival between the histological types did not reach statistical significance.

Vision evaluation was carried out through a detailed subjective assessment of the patient and by clinical examination. In 5 patients, the vision was assessed as non-functional. In all cases, the cause was severe post-radiation damage to the eye. In 61% of the patients, the eye was evaluated as functional. Compared to Imola et al. [11], who has a very consistent approach to orbital reconstruction, our results are slightly worse, but the main cause was the adverse effects of RT. At this point, it should be emphasized again that Imola et al. also included less advanced tumors with invasion into the bony walls of the orbit, which were not included in our cohort of patients.

## Conclusion

Our data support selective orbital preservation in surgical management of patients with sinonasal malignancy that extends through the periorbita. In this study, better survival was observed in the orbital preservation group than in the orbital exenteration group. Overall survival was very similar to other research groups. The high proportion of functional vision is also very encouraging. In recent years, proton radiation therapy has become a trend for SNC, allowing better targeting of the irradiated volume. Thus, further reduction of the adverse radiation effect on critical structures such as the eye bulb and the optic nerve can be assumed. These findings support our approach used for SNC infiltrating the orbit.
